# Enhanced Phenotype Definition for Precision Isolation of Precursor Exhausted Tumor-Infiltrating CD8 T Cells

**DOI:** 10.3389/fimmu.2020.00340

**Published:** 2020-02-27

**Authors:** Amaia Martinez-Usatorre, Santiago J. Carmona, Céline Godfroid, Céline Yacoub Maroun, Sara Labiano, Pedro Romero

**Affiliations:** ^1^Department of Oncology UNIL CHUV, University of Lausanne, Épalinges, Switzerland; ^2^Department of Oncology UNIL CHUV, Ludwig Institute for Cancer Research, University of Lausanne, Épalinges, Switzerland

**Keywords:** tumor-infiltrating CD8 T cells, precursor exhausted T cells, memory-like CD8 T cells, adoptive cell transfer, melanoma

## Abstract

In the context of adoptive T cell transfer (ACT) for cancer treatment, it is crucial to generate *in vitro* large amounts of tumor-specific CD8 T cells with high potential to persist *in vivo*. PD-1, Tim3, and CD39 have been proposed as markers of tumor-specific tumor-infiltrating CD8 T lymphocytes (CD8 TILs). However, these molecules are highly expressed by terminally differentiated exhausted CD8 T cells (Tex) that lack proliferation potential. Therefore, optimized strategies to isolate tumor-specific TILs with high proliferative potential, such as Tcf1+ precursor exhausted T cells (Tpe) are needed to improve *in vivo* persistence of ACT. Here we aimed at defining cell surface markers that would unequivocally identify Types for precision cell sorting increasing the purity of tumor-specific PD-1+ Tcf1+ Tpe from total TILs. Transcriptomic analysis of Tpe vs. Tex CD8 TIL subsets from B16 tumors and primary human melanoma tumors revealed that Tpes are enriched in *Slamf6* and lack *Entpd1* and *Havcr2* expression, which encode Slamf6, CD39, and Tim3 cell surface proteins, respectively. Indeed, we observed by flow cytometry that CD39– Tim3– Slamf6+ PD-1+ cells yielded maximum enrichment for tumor specific PD-1+ Tcf1+ OT1 TILs in B16.OVA tumors. Moreover, this population showed higher re-expansion capacity upon an acute infection recall response compared to the CD39+ counterparts or bulk PD-1+ TILs. Hence, we report an enhanced sorting strategy (CD39– Tim3– Slamf6+ PD-1+) of Tpes. In conclusion, we show that optimization of CD8 TIL cell sorting strategy is a viable approach to improve recall capacity and *in vivo* persistence of transferred cells in the context of ACT.

## Introduction

Sustained antigenic stimulation of CD8 T cells in cancer and chronic infection leads to an exhausted state characterized by high expression of multiple inhibitory receptors, reduced cytotoxicity, and re-expansion capacity (1–3). Far from being a “failed” immune response, this *Tox*-driven exhaustion epigenetic program is essential for the persistence of CD8 T cells in the context of sustained antigenic stimulation (4–6). If CD8 T cells fail to activate the exhaustion program (e.g., when *Tox* is disrupted), persistent antigenic stimulation erodes immunodominant clones by mechanisms such as activation-induced cell death (4, 6).

Recent studies have shown that the pool of exhausted CD8 tumor-infiltrating lymphocytes (TILs) is maintained within the tumor by a precursor subset (memory-like or precursor exhausted, Tpe) that expresses the transcription factor T cell factor 1 (Tcf1), encoded by *Tcf7* (7, 8). Tpe cells can self-renew and differentiate into exhausted cells (Tex). Tex exert (limited) cytotoxic functions while avoiding overstimulation but they lack proliferative potential (8–10).

In the context of adoptive T cell transfer (ACT) for cancer treatment, it is crucial to generate *in vitro* large amounts of tumor-specific CD8 T cells with high potential to persist *in vivo*. To attain tumor-specificity, CD8 T cells are typically isolated from tumor samples. However, not all tumor-infiltrating CD8 lymphocytes (TILs) are tumor-specific (11), and the isolation of tumor-specific TILs is far from trivial. Different studies have shown that tumor-specific TILs can be enriched by means of surface expression of PD-1, Tim3, and CD39 among others (11–15). However, these markers are highly expressed by terminally differentiated Tex, and hence TIL isolation based on these markers enriches in cells with very limited proliferation potential. Therefore, novel strategies to isolate tumor-specific TILs with high proliferative potential, such as Tpe, are needed for improved *in vivo* persistence in ACT. Here we aimed at defining an improved selection strategy to increase the purity of tumor-specific Tpe from total TILs.

## Materials and Methods

### Transcriptomic Analyses

**Figure 1:**
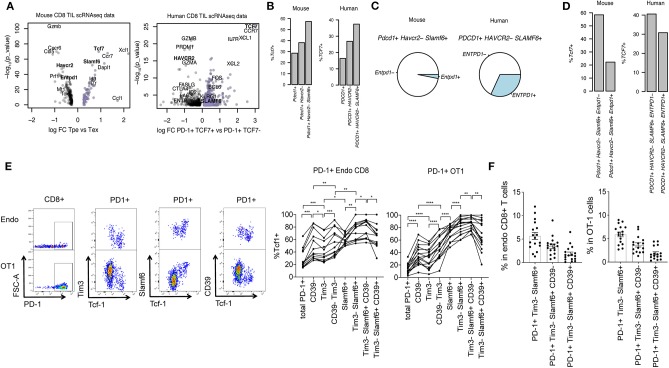
Surface marker identification of PD-1+ Tcf1+ TILs. **(A)** Volcano plots showing differential gene expression between *Pdcd1*+ *Tcf7*+ (encoding PD-1 and Tcf1, respectively) progenitor exhausted (Tpe) and *Pdcd1*+ *Tcf7*– terminal exhausted (Tex) CD8+ T cells. Genes with *p*-value < 0.01 (–log_10_[*p*-value] > 2) are plotted. **(B)** Percentage of *Tcf7*+ cells in *Pdcd1*+, *Pdcd1*+ *Tim3*– or *Pdcd1*+ *Tim3*– *Slamf6*+ CD8+ TILs. **(C)** Pie charts showing mean frequency of *Entpd1*– and *Entpd1*+ (which encodes CD39) cells in total *Pdcd1*+ *Tim3*– *Slamf6*+ CD8+ TILs. **(D)** Percentage of *Tcf7*+ cells in *Pdcd1*+ *Tim3*– *Slamf6*+ *Entpd1*– vs. *Pdcd1*+ *Tim3*– *Slamf6*+ *Entpd1*+ cells. **(A–D)** show analysis of scRNAseq data from murine B16 tumors and human melanoma patients' primary tumors. **(E)** Representative dotplots and quantification of the percentage of Tcf1+ cells in endogenous CD8+ PD-1+ and transferred PD-1+ OT1 cells from B16.OVA tumors before and after subset definition based on the expression of Tim3, Slamf6, and CD39. A repeated measure 1-way ANOVA was performed followed by Tukey's multiple comparison test (*n* = 13–16). Three mice were excluded in the left panel for having acquired <100 endo PD1+ cells. **(F)** Percentage of each marker based subset in total endogenous CD8+ and OT1 cells from B16.OVA tumors (*n* = 16) **(E,F)** show flow cytometry analysis of CD8+ TILs where each dot represents a mouse. Pooled data of two independent experiments.

For the analysis of CD8 TILs melanoma patients we downloaded processed gene expression data [log_2_ (TMP+1)] from NCBI GEO [accession GSE120575, (16)]. Only non-lymph node samples were considered. High-quality cells were filtered based on number of detected genes (between 1,000 and 6,000) and percentage of TPMs mapping to ribosomal genes (<10%) (1482 TILs from 23 patients). PD-1+ CD8 T cells were further filtered from other immune cell infiltrates based on the co-expression of *CD2, CD8A, CD8B, PDCD1* [log_2_ (TMP+1) > = 1], lack of CD4 expression (TPM = 0) and low expression of non-T cell genes *FCER1G, TYROBP, SPI1, IGKC, IGJ, IGHG3* [log_2_ (TMP+1) <3]. Next, we performed differential gene expression analysis between *TCF7*+ [log_2_ (TMP+1) > 0] and *TCF7*- (TPM = 0) cells (cutoff based on the *TCF7* expression distribution of Supplementary Figure 1), using Seurat v2.3.4 and MAST v1.8.2 (17) with parameters min.pct = 0.1, min.diff.pct = 0.01, logfc.threshold = 0.1. For visualization, in the human volcano plot (Figure 1A) we truncated log (fold-change) values at 3 and –log (p-value) at 25. For human TIL virtual gating strategy (Figures 1B–D) we used 1 UMI as a cut-off value to define *PDCD1*+, *HAVCR2*+, *ENTPD1*+ and *SLAMF6*+ cells.

For analysis of murine melanoma CD8 TILs we used processed data (UMI count matrix) from NCBI GEO [accession GSE116390, (18)]. Briefly, gene expression values were normalized and scaled as log(UMI counts / sum UMI count in cell ^*^ 10,000 + 1). Next, differential expression between memory-like/progenitor exhausted subset vs. terminally exhausted subsets [as defined in Carmona et al. (18)] was conducted using Seurat and MAST with parameters min.pct = 0.1, min.diff.pct=0.1, and logfc.threshold = 0.25. For the murine TIL virtual gating strategy (Figures 1B–D), we used 1 UMI as a cut-off value to define *Pdcd1*+, *Havcr2*+, *Entpd1*+, and *Slamf6*+ cells.

### Mice

C57BL/6 (B6) mice were purchased from ENVIGO (Gannat, France) and CD45.1 OT1 mice were bred at the animal facility (Epalinges) of the University of Lausanne. Mice were all females, 7–10 weeks old, and maintained in conventional facilities of the University of Lausanne. This study was approved by the Veterinary Authority of the Swiss Canton of Vaud (authorization VD3360.c) and performed in accordance with Swiss ethical guidelines.

### Melanoma Tumor Model

The B16.OVA cell line was generated as previously described [refers to B16.N4 (19)]. Cells underwent a maximum of two passages from thawing to the time of engraftment and were cultured in complete DMEM (10% FCS (10270-106, Gibco), 1% Penicilin/Streptomycin (15070-063, Gibco), 0.1% β-mercapto ethanol (31350-010, Gibco), 1% Hepes (5-31F00-H, Amimed) in DMEM (31966-021, Gibco).

2 × 10^5^ B16.OVA cells were subcutaneously (s.c.) engrafted on the right flank of B6 mice. After 6 days, 10^5^ CD45.1 OT1 T cells were intravenously (i.v.) transferred. One day later, mice were s.c. vaccinated with 10 μg SIINFEKL (N4) peptide (Protein and Peptide Chemistry Facility, UNIL) and 50 μg CpG (ODN 1826, Microsynth).

Tumors were harvested 21 days post tumor engraftment and dissociated with the Tumor Dissociation Kit (130-096-730, Miltenyi Biotec) following manufacturer's instructions before antibody staining for flow cytometry. Tumors of 6–10 individual mice were pooled before marker based cell sorting and transfer to secondary hosts.

### *Listeria monocytogenes*. OVA Infection

Three hundred flow cytometry-sorted naïve CD8+ CD45.1 cells from spleens of OT1 mice, or 300 OT1 cells (PD-1+, PD-1+ Tim3– Slamf6+ CD39–, or PD-1+ Tim3– Slamf6+ CD39+) isolated from tumors of B16.OVA tumor bearing mice, were i.v. transferred to naïve B6 secondary hosts. The same day, mice were infected i.v. with 2000 colony forming units (cfu) of OVA expressing *Listeria monocytogenes* (Lm-OVA).

Seven days post-infection, spleens from infected mice were collected, smashed through a 70 μm diameter cell strainer (352350, Falcon) and red blood cells were lysed with RBC lysis buffer (158904, Qiagen) before antibody staining for flow cytometry analysis of OT1 cells.

### *Ex vivo* Stimulation of OT1 Cells

Mouse splenocytes were stimulated with 10 μg/ml SIINFEKL peptide or 10 ng/ml PMA (Sigma-Aldrich) and 500 ng/ml ionomycin as positive control (Sigma-Aldrich) for 4 h at 37°C in complete DMEM. Unstimulated cells were used as negative control. BD GolgiPlug and GolgiStop (BD Biosciences) were added to the cells 30 min upon starting the stimulation assay.

### Surface and Intracellular Antibody Staining for Flow Cytometry

Surface antibody staining was performed in darkness for 20 min at 4°C in FACS buffer (2% FCS, 2 mmol/L EDTA in PBS). Cells were stained with the following antibodies: CD3-AF700 (cl. 17A2, eBioscience), CD8-PerCp-Cy5.5 (cl. 53-6.7, eBioscience), CD45.1-BV650 (cl. A20, BioLegend), PD-1-BV711 (cl. 29F-1A12 BioLegend), Tim-3-BV421 (cl. RMT3-23, BioLegend), CD39-PE-Cy7 (cl. Duha59, BioLegend), and Slamf6-APC (cl. 330-AJ, BioLegend). LIVE/DEAD fixable Aqua fluorescent reactive dye (L34957, Thermo Fisher Scientific) or Fixable Viability Dye eFluor 506 (65-0866-14, Thermo Fisher Scientific) was used for dead cell discrimination. For intracellular Tcf1 staining, cells were fixed and permeabilized with Foxp3/Transcription Factor Staining Buffer Set (00-5523-00, eBioscience) according to the manufacturer's instructions and stained for 1 h at 4°C with Tcf-1-Rabbit (2203, Cell Signaling) followed by 15 min Fab aRabbit-PE-TexasRed staining (cl. Poly464, BioLegend), both in Perm/wash buffer. For intracellular cytokine staining, cells were fixed (Fixation buffer; 420801 BioLegend), and permeabilized (Perm/wash buffer; 421002 BioLegend) before being stained with IFNγ-PerCp-Cy5.5 (cl. XMG1.2, eBioscience) and TNFα-APC-Cy7 (cl. MP6-XT22, BioLegend). Samples were analyzed with LSRII flow cytometer (BD Biosciences). For flow cytometry cell sorting, cells were sorted with FACS Aria (BD Biosciences) following the gating strategy from Supplementary Figure 3A and collected in complete DMEM.

### Data and Statistical Analysis

Flow cytometry data were analyzed with FlowJo (TreeStar). Graphs and statistical analysis were made with Prism (GraphPad Software) except scRNAseq data which was analyzed with R (described above). Specific statistical analyses are described in figures' captions. *P*-values are coded as ^*^p > 0.05, ^**^p > 0.01, ^***^p > 0.001, and ^****^p > 0.0001.

## Results

### CD39– Tim3– Slamf6+ PD-1+ CD8 TILs Show the Highest Enrichment for Tcf1+ Cells in Mouse and Human Melanoma Tumors

To identify surface markers of tumor-specific Tcf1+ PD-1+ Tpe that would allow their selection for ACT, we performed a differential gene expression analysis of *Tcf7*+ *Pdcd1*+ (Tpe) vs. *Tcf7*- *Pdcd1*+ (Tex) from scRNAseq data of CD8 TILs from murine B16 (18) and human primary melanoma tumors [publicly available data from Sade-Feldman et al. (16)] (Supplementary Tables 1, 2). In both mouse and human melanoma tumors we observed that *Tcf7*+ *Pdcd1*+ Tpe were enriched in *Slamf6* and depleted of *Havcr2* and *Entpd1*, which encode Slamf6, Tim3, and CD39 cell surface proteins, respectively (Figure 1A). Thus, we hypothesized that the expression patterns of Slamf6, Tim3, and CD39 may help discriminating Tpe from Tex among tumor-specific TILs.

First, we explored the plausibility of this hypothesis *in silico*. A previous report already showed that tumor-specific (identified by tetramer staining) Tim3- Slamf6+ cells were enriched in *Tcf7*+ cells compared to Tim3+ Slamf6- counterparts (7). Here we used *Pdcd1* expression (which encodes for PD-1) as a surrogate marker of tumor specificity (14) and observed that indeed, *Pdcd1*+ *Havcr2*– *Slamf6*+ cells were enriched in *Tcf7*+ cells compared to bulk *Pdcd1*+ or *Pdcd1*+ *Havcr2*– cells (Figure 1B). However, among *Pdcd1*+ *Tim3*– *Slamf6*+ cells we found heterogeneous expression of *Entpd1*, a marker of Tex, both in murine and human melanoma tumors (Figure 1C).

In fact, *Pdcd1*+ *Tim3*– *Slamf6*+ *Entpd1*− cells showed increased enrichment for *Tcf7*+ cells compared to their *Entpd1*+ counterparts (Figure 1D). Thus, at the mRNA level, *Entpd1*– (CD39) *Havcr2*– (Tim3) *Slamf6*+ *Pdcd1*+ cells show the highest enrichment for *Tcf7*+ cells.

Next we sought to verify whether the surface expression of these markers could be used to identify Tpe cells by flow cytometry. To this end, we analyzed Tcf1 expression on total PD-1+ endogenous and transferred OT1 CD8 TILs from B16.OVA tumors before and after marker-based cell selection. We confirmed that CD39– Tim3– Slamf6+ PD-1+ cells showed the highest enrichment for Tcf1+ cells in both endogenous and transferred tumor-specific OT1 cells (Figure 1E), which accounted for the ~3.5% of total endogenous or OT1 TILs (Figure 1F). CCR7 was also highly expressed in Tpes compared to Tex cells (Supplementary Tables 1, 2) but antibody staining of this chemokine receptor was poor and did not select Tcf-1+ CD8 T cells in B16.OVA tumors (Supplementary Figure 2A).

In conclusion, selection of CD39– Tim3– Slamf6+ PD-1+ TILs by surface antibody staining allows for maximum enrichment of Tcf1+ tumor-specific Tpe.

### CD39– Tim3– Slamf6+ PD-1+ CD8 TILs Show Increased Re-expansion Capacity and Functionality Compared to CD39+ Counterparts or Bulk PD-1+ TILs

**Figure 2:**
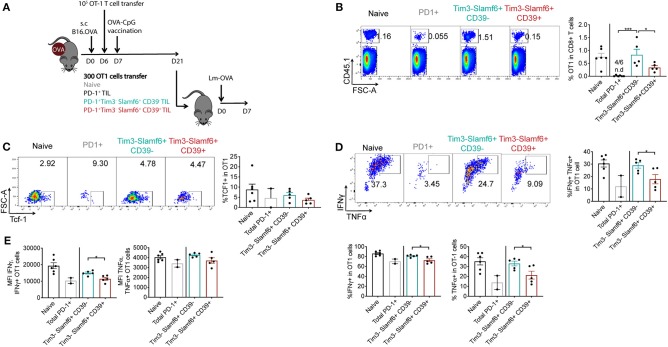
Re-expansion capacity assessment of PD-1+ TIL subsets in Lm-OVA infection. **(A)** Experimental design of panels B-E. Briefly TILs of 6 B16.OVA tumors (21 days-post engraftment) were pooled before marker based cell sorting (Supplementary Figure 3A) and 300 isolated cells were transferred to each recipient B6 mice (*n* = 5–6/group). The same day recipients were infected with 2000 cfu Lm-OVA. **(B)** Representative dotplots and quantification of the percentage of transferred OT1 cells in total CD8+ T cells following the gating strategy of Supplementary Figure 3B. n.d: non-detected. Representative of two independent experiments. **(C)** Representative dotplots and quantification of Tcf1+ cells in total transferred OT1 cells. **(D)** Representative dotplots and quantification of the percentage of IFNγ+, TNFα+ and double IFNγ+ TNFα+ cells in total transferred OT1 cells following the gating strategy of Supplementary Figure 3C. **(E)** IFNγ median fluorescent intensity (MFI) and TNFα MFI in IFNγ+ and TNFα+ transferred OT1 cells respectively. **(B–E)** show analysis of transferred OT1 cells from spleens of secondary hosts 7 days after Lm-OVA infection. **(D,E)** Show *ex vivo* analysis of OT1 cells restimulated with SIINFEKL peptide. A 1-way ANOVA followed by Tukey's multiple comparison test in panel B and an unpaired *t*-test between mice receiving CD39− vs. CD39+ Tim3− Slamf6+ PD1+ OT1 cells in panels **(D,E)** was performed. Bars represent the mean ± SEM and each dot a mouse (*n* = 2–6/group).

Tpe cells have been described as those cells that maintain antigen-specific responses in the context of chronic infection and tumors (8, 9). Therefore, highly homogenous Tpe cell products would be ideal in the context of ACT to improve persistence of transferred cells in the hosts. Thus, we investigated whether our newly described CD39– Tim3– Slamf6+ PD-1+ TIL subset display better recall responses and persistence *in vivo* compared to bulk TIL populations. To this end, either 300 PD-1+, PD-1+ Tim3– Slamf6+ CD39–, or PD-1+ Tim3– Slamf6+ CD39+ OT1 CD8 TILs cells were isolated from B16.OVA tumors 21 days post-tumor engraftment following the gating strategy of Supplementary Figure 3A and transferred to a secondary host that was infected on the same day with OVA-expressing *Listeria monocytogenes* (Lm-OVA). Naïve OT1 cells were also transferred as control (Figure 2A). Seven days post-infection (PI), we observed increased frequencies of transferred OT1 cells in mice receiving PD-1+ Tim3– Slamf6+ CD39– cells compared to those receiving their CD39+ counterparts or total PD-1+ OT1 CD8 TILs (Figure 2B), indicating their enhanced recall response and persistence. At 7 days PI, most transferred cells were Tcf1– (Figure 2C), consistent with previous reports showing that tumor-specific Tcf1+ cells differentiate into Tcf1– negative cells (7, 8).

In addition to the enhanced expansion capacity, PD-1+ Tim3– Slamf6+ CD39– cells showed enhanced functionality in the recall response compared to their CD39+ counterparts, with increased frequency in IFNγ, TNFα and IFNγ+TNFα double expressing cells (Figure 2D) as well as increased expression of these molecules (Figure 2E).

Altogether, selection of CD39– Tim3– Slamf6+ cells among PD-1+ TILs led to an enhanced persistence and functionality of transferred TILs in a recall response compared to their CD39+ counterparts or bulk PD-1+ TILs.

## Discussion

ACT therapy of *in vitro* expanded TILs has shown promising clinical efficacy in melanoma patients (20). However, poor *in vivo* persistence of transferred cells remains a major limitation. TIL culture conditions can be optimized to obtain cell products with longer *in vivo* persistence (21–23). However, these protocols bypass selection of tumor-specific T cells, limiting the anti-tumor capacity of expanded T cells. Here we propose a flow cytometry gating strategy that enables the selection of tumor-specific TILs with higher *in vivo* persistence and functionality in recall responses compared to bulk populations.

In the tumor, high affinity T cells show increased PD-1 expression compared to low affinity counterparts (19). Therefore, isolation of PD-1+ cells may allow selection of high affinity T cells, which show enhanced tumor control than low affinity T cells (19). Moreover, high affinity T cells may include those recognizing mutated self-antigens (neoantigens) which have been shown to respond to vaccination (24, 25) and anti-PD-1 therapy (24).

We propose the use of PD-1 as a marker of tumor specificity as previously described (14) while simultaneously gating out Tim3+ and CD39+ cells. Even though Tim3 and CD39 can further enrich in tumor-specific TILs (11, 13, 15), this is achieved at the expense of a dramatic loss of *in vivo* recall capacity. Recent insights into the heterogeneity of tumor antigen-specific CD8 TILs revealed the existence of a small subset of memory-like PD-1+ CD8 TIL with high proliferation potential and self-renewal capacity, the Tpes.

By transcriptomic and flow cytometry analyses of CD8 TILs from murine B16 and human primary melanoma, we found that Tpes can be efficiently captured by gating on Tim3- Slamf6+ CD39– cells. Indeed, this CD8 T cell subset yielded maximum enrichment for Tcf1+ cells among PD-1+ TILs. Furthermore, adoptively transferred CD39– Tim3– Slamf6+ TILs exhibited increased persistence and functionality in recall responses to the Lm-OVA acute infection, compared to the CD39+ counterparts. This is consistent with previous reports associating CD39 with terminally differentiated exhausted CD8 T cells (16, 26, 27). Previous reports (28–30) described CXCR5 as a marker of CD8 T cells with stem-cell like properties as Tpes. However, in the analyzed mouse and human melanoma RNAseq datasets CXCR5 was not enriched in Tpes (Supplementary Tables 1, 2), neither allowed identification of Tcf1+ cells by flow cytometry in B16.OVA tumors (Supplementary Figure 2B). Thus, the ability of CXCR5 staining to identify cells with stem-cell like properties may be context dependent.

Hence, we conclude that our proposed gating strategy represents an optimal trade-off between enriching for tumor-specific TILs with high-affinity and preserving recall capacity.

Altogether, we show that selection of CD39– Tim3– Slamf6+ PD-1+ TILs enables enrichment of tumor specific Tcf1+ PD-1+ TILs Tpe that may enhance efficacy of adoptive cell transfer therapies in cancer patients.

## Data Availability Statement

The datasets generated for this study are available on request to the corresponding author.

## Ethics Statement

The animal study was reviewed and approved by Veterinary Authority of the Swiss Canton of Vaud (authorization VD3360.c).

## Author Contributions

AM-U, SC, SL, and PR: conception and design. AM-U and SC: development of methodology. AM-U, SL, CG, and CY: acquisition of data. AM-U, SC, and SL: analysis and interpretation of data. AM-U, SC, SL, CG, and PR: writing review and/or revision of the manuscript. SC and PR: study supervision.

### Conflict of Interest

PR receives speaker honoraria from Bristol-Myers Squibb and Roche and is the recipient of research grants in immune-oncology from Roche pRED, Zurich, CH. The remaining authors declare that the research was conducted in the absence of any commercial or financial relationships that could be construed as a potential conflict of interest.
