# Supplementary material for: Enhanced Phenotype Definition for Precision Isolation of Precursor Exhausted Tumor-Infiltrating CD8 T Cells
Source: Front Immunol. 2020 Feb 27;11:340. doi: 10.3389/fimmu.2020.00340 (PMC7056729; doi:10.3389/fimmu.2020.00340)
Supplement: Supplementary file 1 [file Data_Sheet_1.PDF]

Supplementary Materials for

Enhanced phenotype definition for precision isolation of precursor exhausted tumor-infiltrating CD8 T cells

Amaia Martínez-Usatorre[1,3], Santiago J. Carmona[2], Céline Godfroid[1], Céline Yacoub Maroun[1], Sara Labiano[1], Pedro Romero[1*]

Correspondence to: pedro.romero@unil.ch

This PDF file includes

Supplementary figure 1: Histogram of Tcf7 expression for the mouse and human datasets.

Supplementary figure 2: Representative dotplots of CCR7 and CXCR5 staining.

Supplementary figure 3: Representative gating strategies used for Figure 2.

Supplementary table 1: Differential gene expression of mouse Tpe vs Tex (separate .xlsx)

Supplementary table 2: Differential gene expression of human Tpe vs Tex (separate .xlsx)

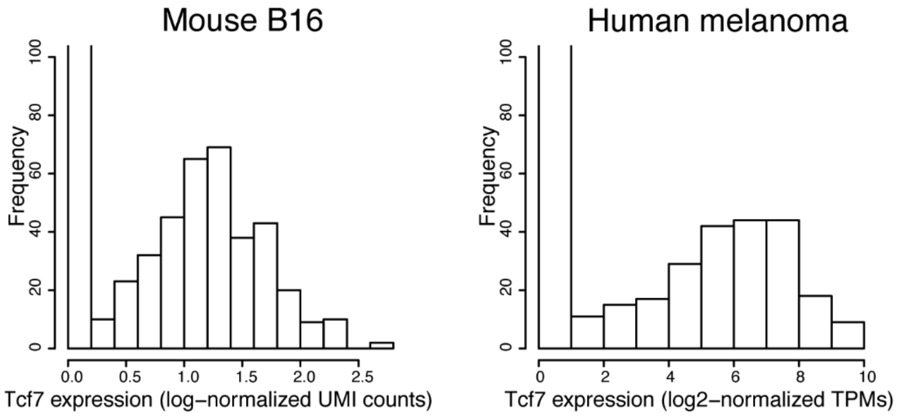

Supplementary figure 1: Histogram of Tcf7 expression for the mouse dataset (left) with log-normalized UMI counts on the x axis and human dataset (right) with log2-normalized TPM values.

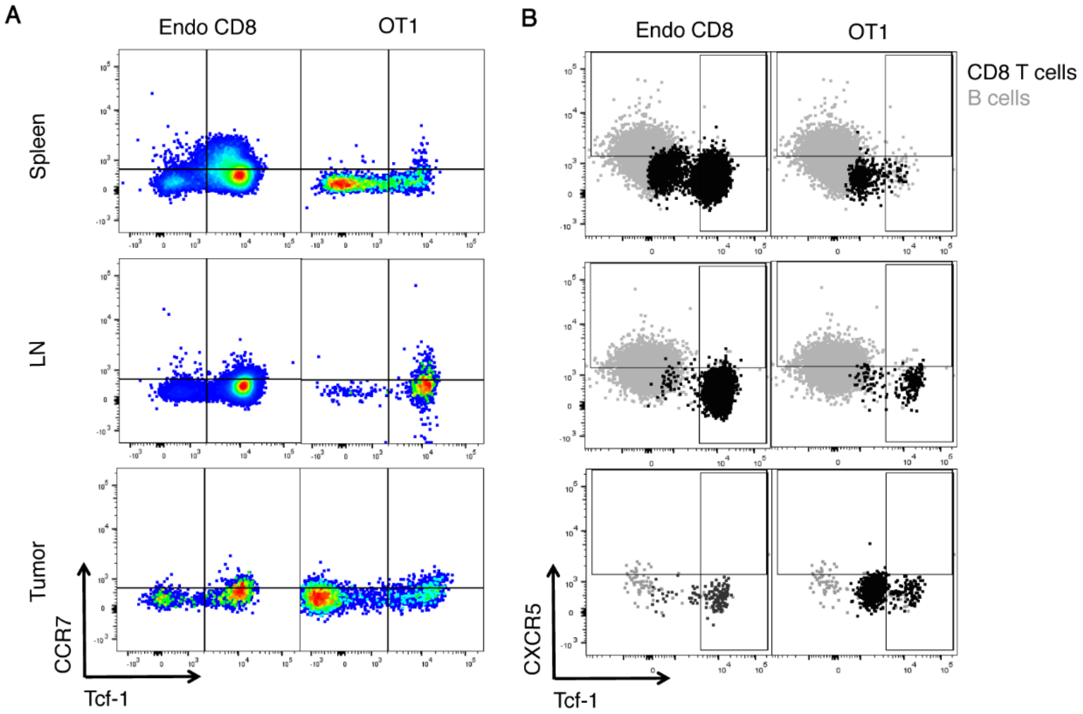

Supplementary figure 2: Representative dotplots of CCR7 and CXCR5 staining of endogenous CD8+ and transferred OT1 cells. Spleens, draining lymph nodes (LN) and B16.OVA tumors were harvested and dissociated 21 days post-tumor engraftment, 14 days post-vaccination with OVA-CpG as described in material and methods. **A.** CCR7 (cl 4B12, 120115 Biolegend) staining was performed at 37°C for 30’. **B.** CXCR5 staining was performed in 3 steps as previously described (Im et al. 2016). An unlabeled anti-mouse CXCR5 Rat IgG2a (cl 2G8, 551961 BD Biosciences) was used as primary antibody, a biotinylated goat-anti-Rat antibody as secondary and BV421 conjugated streptavidin as tertiary. The 3-step staining was performed before addition of other antibodies. Stainings were performed on ice for 20’. In black endogenous CD8+ (left panels) and transferred OT1 cells (right panels) are shown and in grey B cells as positive control of CXCR5 staining. Data shows representative dotplots of n=16 (A) n=27 (B) tumors.

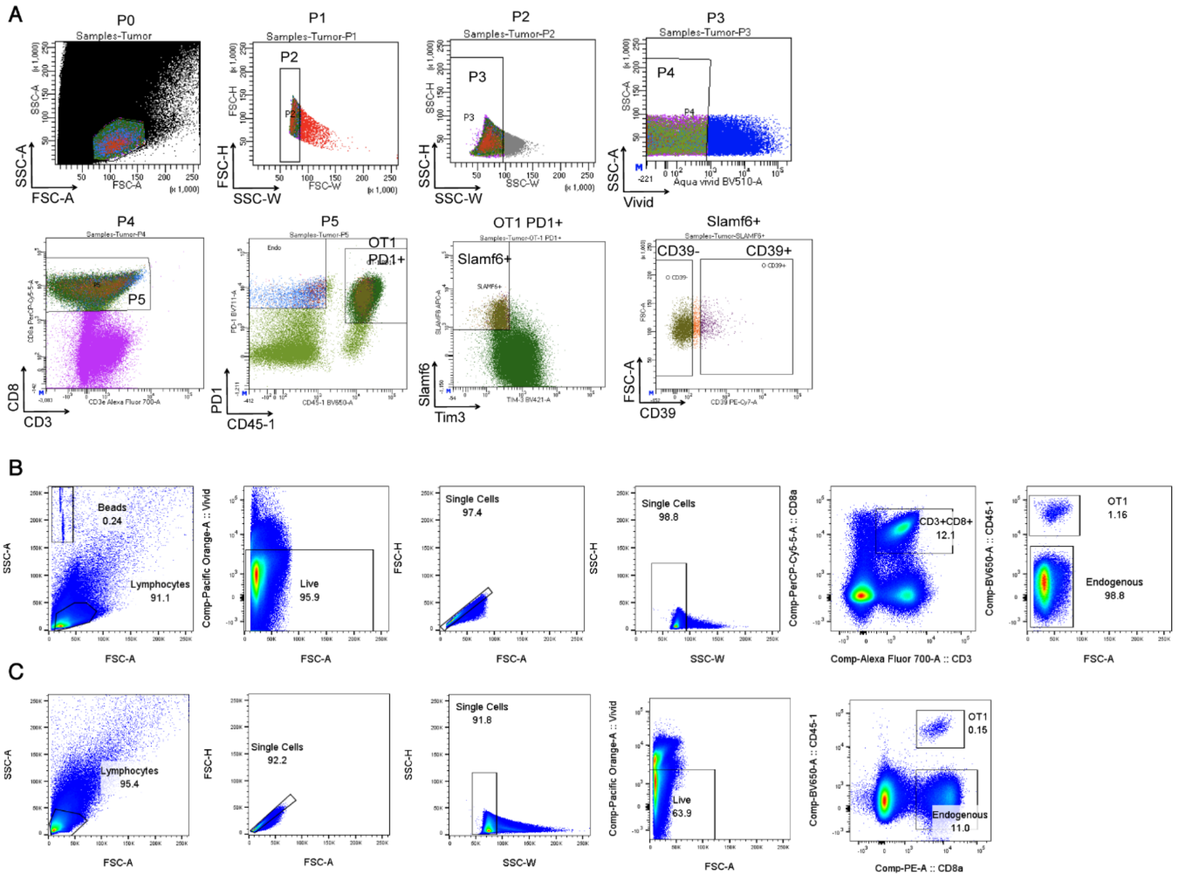

Supplementary figure 3: A. Representative gating strategy used to isolate the different marker based OT1 populations from B16.OVA tumors 21 days post-tumor engraftment (Figure 2A). **B.** Representative gating strategy used to identify transferred OT1 cells in secondary hosts 7 days post-infection for figures 2B and 2C. **C.** Representative gating strategy used to identify transferred OT1 cells in secondary hosts 7 days post-infection for figure 2D.
